# Hemodynamics in Left-Sided Cardiomyopathies

**DOI:** 10.31083/j.rcm2512455

**Published:** 2024-12-24

**Authors:** Guido Del Monaco, Francesco Amata, Vincenzo Battaglia, Cristina Panico, Gianluigi Condorelli, Giuseppe Pinto

**Affiliations:** ^1^IRCCS (Istituto di Ricerca e Cura a Carattere Scientifico) Humanitas Research Hospital, 20089 Rozzano-Milan, Italy; ^2^Department of Biomedical Sciences, Humanitas University, 20072 Pieve-Emanuele-Milan, Italy

**Keywords:** cardiomyopathies, hemodynamics, pressure-volume relationship, cardiac catheterization

## Abstract

Cardiomyopathies, historically regarded as rare, are increasingly recognized due to advances in imaging diagnostics and heightened clinical focus. These conditions, characterized by structural and functional abnormalities of the myocardium, pose significant challenges in both chronic and acute patient management. A thorough understanding of the hemodynamic properties, specifically the pressure-volume relationships, is essential. These relationships provide insights into cardiac function, including ventricular compliance, contractility, and overall cardiovascular performance. Despite their potential utility, pressure-volume curves are underutilized in clinical settings due to the invasive nature of traditional measurement techniques. Recognizing the dynamic nature of cardiomyopathies, with possible transitions between phenotypes, underscores the importance of continuous monitoring and adaptive therapeutic strategies. Enhanced hemodynamic evaluation can facilitate tailored treatment, potentially improving outcomes for patients with these complex cardiac conditions.

## 1. Introduction

Cardiomyopathies encompass a diverse spectrum of cardiac disorders characterized by structural and functional abnormalities of the myocardium, leading to impaired systolic and/or diastolic function. Among these, left-sided cardiomyopathies present a significant clinical challenge due to their varied phenotypes, including hypertrophic, restrictive and dilated patterns [1, 2].

Understanding the intricacies of pressure-volume relationships (PVR) and hemodynamics is essential in managing patients with left-sided cardiomyopathies. These curves provide valuable insight into myocardial contractility, ventricular compliance and overall cardiac performance. Notably, variations in preload, afterload, and contractility significantly influence these curves, necessitating a nuanced approach in their interpretation and clinical application [3, 4]. Moreover, the therapeutic landscape in left-sided cardiomyopathies is continuously evolving, with several pharmacological agents and interventional strategies aimed at modulating cardiac function and structure [5]. The impact of these interventions on PVR underscores the importance of comprehensively assessing hemodynamic parameters to tailor treatment strategies to individual patient needs.

The aim of this review is to analyze the dynamic interplay between pressure-volume curves and the pathophysiology of different left-sided cardiomyopathy phenotypes. We explore how variations in volume status, afterload, and therapeutic interventions shape these curves, ultimately impacting clinical outcomes and guiding optimal management approaches.

### PVR 

PVR is created by concurrently measuring ventricular pressure and volume and plotting them on the same plane. It is a valuable method for assessing heart failure (HF) and pulmonary hypertension (PH) hemodynamics. End-systolic pressure-volume relationship (ESPVR) and end-diastolic pressure-volume relationship (EDPVR) derived from PVR measurement have been studied as important indicators of performance of the heart. These indices are useful for evaluating intrinsic ventricular contractility and distensibility independently of preload [6]. In clinical practice, it is preferable to estimate multiple PVR using a conductance catheter in the ventricle and alter the preload by occluding the inferior vena cava or employing other techniques such as the multiple-beat method [7].

## 2. Hypertrophic Cardiomyopathy (HCM)

HCM is a relatively common inherited heart disease with diverse and complex phenotypic presentation, genetic expression and clinical course [8]. The estimated prevalence is 1:500 in the general population based on the disease phenotype, and higher (1:200) accounting for familial transmission, subclinical cases, and pathogenic sarcomere mutations [9]. Contemporary treatments and interventions, personalized to target adverse pathways, have significantly reduced HCM mortality >10-fold from 6%/year reported early on to 0.5%/year, currently one of the lowest of all major disease-related risks to living [10]. With currently available management options, mortality specifically attributable to HCM is now distinctly uncommon and largely limited to a minority of nonobstructive patients with progressive refractory HF [11, 12].

Obstructive HCM is defined as the presence of left ventricular outflow tract (LVOT) obstruction, i.e., the presence of a gradient ≥30–50 mmHg, occurring at rest or with Valsalva maneuver or physical provocation (exercise stress echocardiography) [13]. As recommended in the last ESC guidelines on cardiomyopathies, pharmacological provocation with inotropic agents (as dobutamine) is not advised, because it is not physiological and can be poorly tolerated [1]. As shown by Losi *et al*. [14], dobutamine stress test in a group of HCM patients induced new wall motion abnormalities and a global depression of left ventricular (LV) systolic function without provoking LVOT gradient. This phenomenon may occur due to an increase in heart rate (HR) or because of microvascular dysfunction [14].

HCM is characterized by abnormal thickening of the heart muscle which can alter heart function, affecting both the filling and ejection phases of the cardiac cycle. These alterations can be visualized and analyzed through pressure-volume curves, which represent a powerful diagnostic tool for assessing cardiac function [3].

### 2.1 PVR in HCM 

**Fig. 1.**
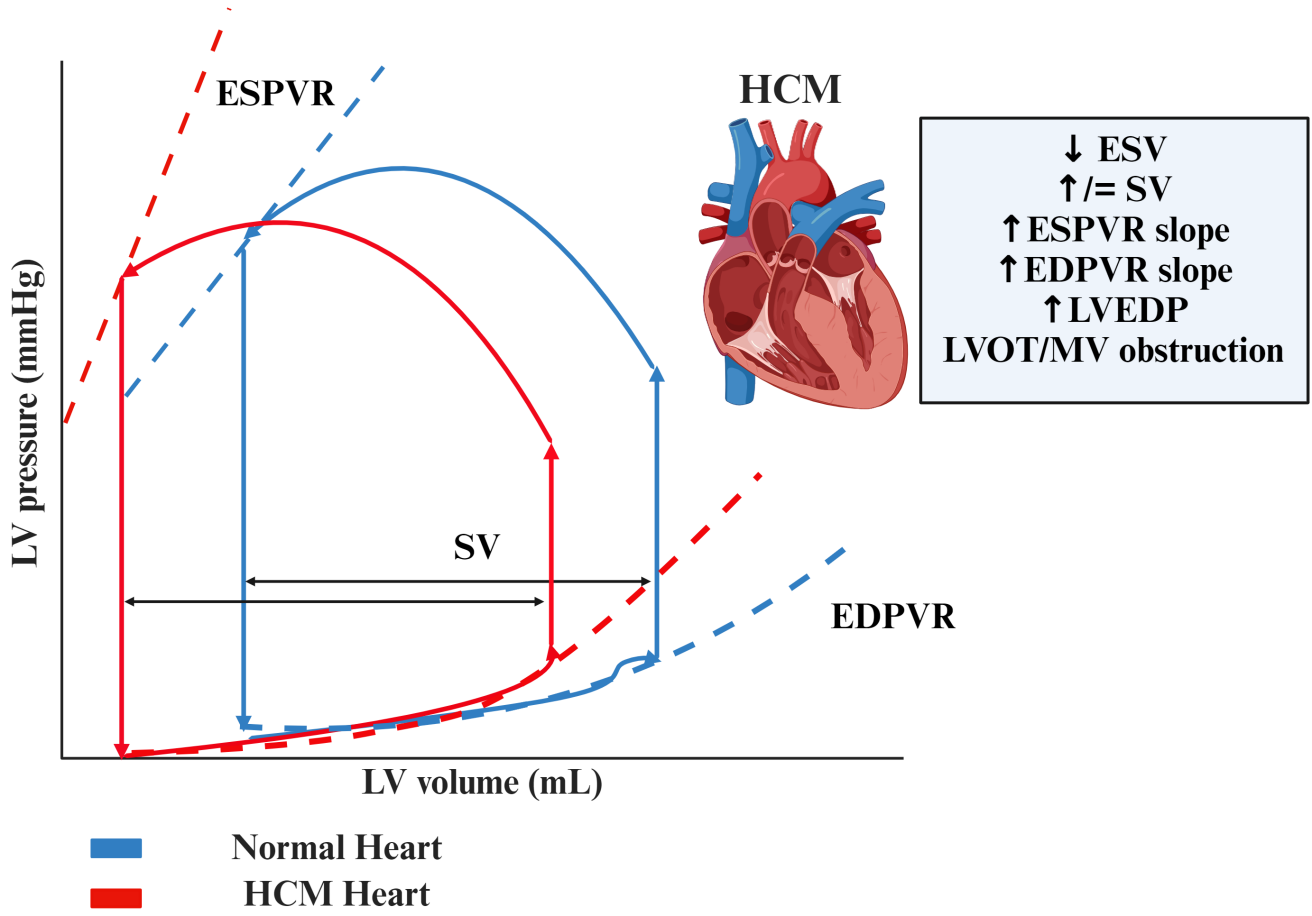
**Pressure-volume loops in HCM compared to normal hearts**. Abbreviations: EDPVR, end-diastolic pressure-volume relationship; ESPVR, end-systolic pressure-volume relationship; ESV, end-systolic volume; HCM, hypertrophic cardiomyopathy; LV, left ventricle; LVEDP, left ventricular end-diastolic pressure; LVOT, left ventricular outflow tract; MV, mid ventricular; SV, stroke volume. ↑ increase, ↓ decrease, = no change.

PVR in patients with HCM may exhibit various anomalies compared to healthy subjects. These findings have been demonstrated using hemodynamic, contrast angiographic and echocardiographic techniques (Fig. 1).

#### 2.1.1 Increased Slope and Leftward Shift of ESPVR

This reflects the heart’s increased ability to generate pressure due to muscle thickening, especially during the first stages of the disease when the heart is hypercontractile. This phenomenon is mainly caused by a reduction in end-systolic volume (ESV), while end-diastolic volume (EDV) is normal or mildly reduced, with a consequent increase in stroke volume (SV). The increased contractility in patients with HCM is expressed by a steep ESPVR and a consequently increased end-systolic elastance (Ees), which is shifted leftwards and reaches values that are 4 to 5 times higher than normal hearts.

#### 2.1.2 Increased Slope of EDPVR

Patients with HCM often exhibit decreased ventricular compliance, making the heart less capable of filling effectively at low pressures. This is represented by a steeper EDPVR, indicating increased left ventricular end-diastolic pressure(LVEDP) for relatively low volumes.

#### 2.1.3 Elevated LVEDP

In HCM hearts, due to chamber stiffness and prolonged isovolumetric relaxation time (IVRT), LVEDP are higher than normal hearts, mainly during exercise, reflecting an increased pulmonary capillary wedge pressure (PCWP) and thus symptoms of pulmonary congestion [4].

#### 2.1.4 Reduction in EDV 

Due to muscle thickening, the internal volume of the ventricle may be reduced, limiting the amount of blood the heart can pump with each beat. This feature may be more pronounced in patients with restrictive physiology in the advanced phases of the disease [15].

#### 2.1.5 Reduction in ESV

In HCM, ESV is reduced, particularly in the early stages when the heart exhibits hypercontractility leading to a more efficient ejection of blood during systole, thus resulting in a reduced ESV.

#### 2.1.6 Potential Presence of Intraventricular Pressure Gradient 

In some patients with obstructive HCM, a significant pressure gradient can develop between the ventricle and the aortic outflow during systole due to blood flow obstruction. Around one-third of patients with HCM present dynamic obstruction at rest, and another one-third present with inducible obstruction during stress [16]. LVOT obstruction is dynamic in nature and could be augmented by any process leading to alterations in left ventricular loading conditions or myocardial contractility. Indeed, LVOT gradient variability has been associated with fluctuations in volume status, autonomic nervous activity, diurnal variation, pharmacotherapy, exercise, general anesthesia, conscious sedation, recent cardioplegia, and even physical positioning during gradient assessment [17]. Apart from the typical pressure drop across LVOT, cardiac catheterization in patients with advanced dynamic obstruction may also disclose the Brockenbrough–Braunwald–Morrow sign [18, 19]. This sign is characterized by a decrease in arterial pulse pressure after a premature ventricular contraction: the post-extrasystolic potentiation, reflected by a transient increase in intraventricular pressure following a premature ventricular contraction, is not propagated through the LVOT because of the concomitant greater dynamic obstruction, so that the LVOT and the aortic post-extrasystolic pressures do not increase. They assumed that the mechanism for this phenomenon was the dynamic narrowing of the LVOT secondary to increased contraction of the hypertrophied muscle. The Brockenbrough–Braunwald–Morrow sign was typically described after ventricular premature beats but it is also present after atrial premature beats [20]. In HCM flow obstruction could also occur at the middle of the left ventricle, the so-called midventricular obstruction (MVO). MVO is a distinct and recognized phenotype of HCM, occurring as a result of segmental mid-septal hypertrophy and hypercontractility of the lateral ventricular wall along with the misplacement of the hypertrophied papillary muscles. MVO has been demonstrated as an independent predictor of sudden death and associated lethal arrhythmic events as well as a determinant of progression to end-stage HCM and HF-related death [21].

#### 2.1.7 Hemodynamics in HCM During Exercise

Patients with HCM usually display symptoms like poor exercise tolerance, chest pain and dyspnea during efforts. As shown by Takahashi *et al*. [22], compared to healthy controls, HCM patients present a decrease of left ventricular ejection fraction (LVEF) at peak exercise (instead of an increase) and a reduced increase in cardiac output (CO), mainly due to a reduced or unchanged SV. Moreover, pulmonary diastolic pressure during exercise increased to a greater extent than in healthy controls [22]. Consequently, PVR during exercise is shifted rightwards and downwards. Therefore, HCM patients during physical exercise show an impaired LV functional reserve, mainly due to a reduction in SV reserve, while symptoms of pulmonary congestion may partially depend on elevated diastolic filling pressures [22].

#### 2.1.8 End-Stage HCM (ES-HCM)

Beyond the traditional HCM phenotype previously described, Olivotto *et al*. [23] proposed a four-stage classification for the clinical course of HCM. These phases are represented by: stage I or “non-hypertrophic HCM”, characterized by absence of overt LV hypertrophy; stage II, or “classic HCM”, in which LV is hypertrophic and hypercontractile (LVEF usually >65%) in the absence of signs of fibrosis or disease progression; stage III or “adverse remodeling”, which is defined by the presence of fibrosis with worsening LV function (LVEF 50–65%) without signs of hemodynamic decompensation; stage IV, or “overt dysfunction”, characterized by severe functional deterioration of LVEF (<50%) and signs and symptoms of HF [23]. This corresponds to the so-called “ES-HCM” and is considered an uncommon complication of the disease [24]. Its pathophysiological mechanism has not been fully clarified, but may involve myocardial ischemia and fibrosis which can evolve finally in left ventricular remodeling and severe left atrial dilation. ES-HCM is associated with hemodynamic changes, decompensation and therefore with adverse clinical outcome. The annual mortality exceeds 10% and more than 60% of patients die or undergo heart transplantation [25]. Currently, two main morpho-functional manifestations of ES-HCM have been described. The first one has been defined as hypokinetic-restrictive and is characterized by severe diastolic dysfunction, LV stiffness and mild-to-moderate LVEF impairment. Sometimes, certain residuals of asymmetrical LV hypertrophy can be present but the distinction with primary restrictive cardiomyopathy is challenging. The second one is the so-called hypokinetic-dilated form, which is defined by the presence of LV dilation and spherical remodeling with or without signs of residual left ventricular hypertrophy [23]. As for the restrictive phenotype of ES-HCM, a distinction from primary dilated cardiomyopathy may be difficult. Hemodynamics, and therefore PVR in these two subtypes reflect those of the respective cardiomyopathy (see the dedicated sections 3 and 4 for description).

#### 2.1.9 Atrial Fibrillation (AF) and Left Atrium (LA) in HCM

AF is the most common sustained arrhythmia in HCM patients and its estimated prevalence is about six times greater than in the general population, with an annual incidence of 2–4% [26]. Up to a quarter of HCM patients will develop clinical AF during the course of the disease. This risk of developing AF increases with age (especially >60 years), while, if considering even subclinical AF episodes, the estimated prevalence is nearly 50% [27]. Traditionally, AF onset in HCM has been regarded as a major complication, which represents a turning point and marker for increased mortality, morbidity and worsening quality of life due to an increase of HF episodes and systemic thromboembolism [28, 29]. Hemodynamically, the elevated ventricular rates and the loss of contribution of atrial contraction to left ventricular filling is often poorly tolerated by a stiff, hypertrophied and noncompliant LV reflecting in a reduction of left ventricular end-diastolic volume (LVEDV) and therefore of SV. Poor diastolic filling may be detrimental especially in presence of LVOT obstruction (LVOTO). Moreover, LA emptying may be difficult and therefore may lead to a rise of PCWP [30], causing symptoms like dyspnea, palpitations and poor effort tolerance. Acute decompensation due to elevated mean ventricular response, requiring external electrical cardioversion and hemodynamic support, is not a common condition in HCM patients. The pathophysiological mechanisms which lead to AF development in HCM can be synthetized from three main determinants: (1) LA enlargement, traditionally considered as the strongest one, which is driven by an increase in LA pressure secondary to diastolic dysfunction and mitral regurgitation (MR) (mainly in obstructive HCM (OHCM)) [31, 32]; (2) primary atrial myopathy, which is present in up to 20–30% of patients and is represented by extensive fibrosis in the absence of overt secondary cause without LA enlargement [33]; (3) comorbidities traditionally linked to AF pathophysiology, such as arterial hypertension, diabetes, cigarette smoke and obesity [30].

Currently, AF management in HCM includes: prevention of thromboembolism with anticoagulation (preferably with direct oral anticoagulants), which should be initiated after the first AF episode despite stroke risk scoring systems that are not informative in HCM patients; and rate or rhythm control with a priority in maintenance of sinus rhythm with antiarrhythmic drugs [34]. Moreover, catheter or surgical ablation has been shown to be effective in decreasing AF episodes and symptom burden, albeit with a lower success rate compared to the outcomes observed in non-HCM populations [35, 36].

### 2.2 Pharmacological Therapy of HCM and Changes in Pressure-Volume (PV) Loops

The hemodynamic effects of drugs currently used for HCM management are displayed in Table 1.

**Table 1.** **Hemodynamic effects of pharmacological therapy in HCM**.

	BB	CCB	Disopyramide	Cardiac myosin inhibitors
EDV	↑	↑	↔	↑
SV	↑	↔	↔/↓	↑
LVOT gradient	↓ (++ during exercise)	↓/↔	↓ (++ at rest)	↓
HR	↓	↓/↔	↔	↔
Mitral regurgitation	↓	↔	↓	↓
ESPVR slope	↓	↓	↓	↓
EDPVR slope	↓/↔	↓	↔/↓	↓
LVEDP	↔	↔/↑	Not investigated	↓

Abbreviations: BB, beta-blockers; CCB, calcium-channel blockers; EDPVR, end-diastolic pressure-volume relationship; EDV, end-diastolic volume; ESPVR, end-systolic pressure-volume relationship; HR, heart rate; LVEDP, left ventricular end-diastolic pressure; LVOT, left ventricular outflow tract; SV, stroke volume; HCM, hypertrophic cardiomyopathy. ↑ increase, ↓ decrease, ↔ no change, ++ more pronounced.

#### 2.2.1 Beta-Blockers (BB)

BB play a key role in HCM management by modulating heart rate and myocardial contractility, leading to improved diastolic filling and symptom relief, particularly in patients with obstruction. Despite their efficacy, careful dosing and monitoring are necessary due to potential side effects such as bradycardia and hypotension. The introduction of BB was based on early studies documenting that an increase in heart rate was accompanied by a reduction in LV volume, and a subsequent exacerbation of the LVOT obstruction [37]. The beneficial effects of BB have been the focus of numerous studies, which showed that this class of drugs (mainly first-generation BB such as propranolol) was effective in decreasing LVOT gradient, mainly during exercise or isoprenaline infusion [38, 39] with a theoretical benefit in improving symptoms and exercise capacity. Unfortunately, they don’t seem to change the natural course of the disease [40] neither are they able to reduce oxygen consumption [41]. More recently, metoprolol was shown to increase stroke volume at rest and peak exercise following changes in end-diastolic volume, LVOT gradient, and degree of mitral regurgitation [42] with no benefit in reducing LVEDP rise during exercise.

#### 2.2.2 Calcium-Channel Blockers (CCB)

CCB are traditionally considered a cornerstone in the management of HCM by exerting their effects on myocardial contractility and relaxation. However, their use is often limited due to individual patient response variability and potential adverse effects [43]. Nonetheless, they remain an important therapeutic option in the management of HCM, particularly in patients with obstruction and symptoms which are refractory to other treatments and/or in cases of BB contraindication/intolerance. Acute intravenous administration of verapamil has been shown to decrease LVOT obstruction in patients with HCM but the increase in exercise capacity does not necessarily correlate with the decrease in subvalvular gradient [44]. Additionally, Verapamil improves left ventricular diastolic filling reducing IVRT, without altering systolic function [45]. Moreover, it increases EDV and ventricular compliance [46]. This drug, however, has the potential for serious electrophysiological and mechanical complications, like atrio-ventricular block, excessive vasodilation and severe contractile depression [47]. This problem has prompted investigators to test the efficacy of other CCB. Nifedipine, because of its potent vasodilating effect, may worsen obstruction, and its effects on diastolic properties are controversial, mainly in patients with normal PCWP [48]. Diltiazem has been shown to be effective in improving diastolic function by both direct action and by causing changes in hemodynamics and loading conditions without increasing LVOT gradient but with a potentially harmful elevation in LVEDP [49].

#### 2.2.3 Disopyramide

Disopyramide is a class Ia antiarrhythmic drug, historically used for the treatment of supraventricular arrhythmias. However, its contemporary use is often reserved for patients with HCM who are persistently symptomatic despite BB or CCB treatment and have evidence of LVOT obstruction [50, 51]. The pharmacological rationale for using disopyramide is largely based on its strong negative inotropic property, which is not explained by a direct action on the myofilaments or in calcium sensitivity but in reducing the amplitude of calcium and late sodium currents. In patients with obstructive HCM, the beneficial effect of disopyramide is on LV contractility, specifically by decreasing early LV ejection flow acceleration [52]. The effect on the PV loop is therefore a reduction in ESPVR slope with a modest reduction of global systolic function. In obstructive HCM, where ejection time is prolonged due to LVOT obstruction, disopyramide reduces ejection time. Moreover, the effect of disopyramide in reducing LVOT gradient seems to be more effective than other drugs (like BB and CCB) and is more pronounced in reducing rest LVOT gradient. The effect of disopyramide on LV volumes seems to be very limited and is different from normal hearts where it can decrease LVEF [53], meaning its reduction in HCM hearts is not so relevant. Finally, by reducing the diastolic calcium transient and sarcoplasmic reticulum calcium overload, disopyramide may have a potential role in improving diastolic properties of HCM myocardium by reducing EDPVR slope and therefore increasing compliance [54, 55]. The role of disopyramide in varying LVEDP and PCWP has not been clearly investigated. The main issues linked to this drug are appropriate dosing and monitoring of disopyramide to mitigate the potential for anticholinergic adverse events and corrected QT (QTc) interval prolongation.

#### 2.2.4 Cardiac Myosin Inhibitors 

Cardiac myosin inhibitors are a novel therapy for HCM that act by targeting the underlying molecular mechanism of HCM which is characterized by hypercontractility, impaired relaxation and altered myocardial energetics. By inhibiting the excessive myosin-actin interactions, these drugs reduce myocardial contractility (and therefore the slope of ESPVR) and promotes a relaxed state of myosin which subsequently improves diastolic function by increasing LV compliance (reducing EDPVR slope) [56]. This modulation of cardiac function leads to alterations in the PVR in HCM patients. The principal effects of these drugs on HCM are a decrease in peak pressures and a shift of PVR toward higher volumes and higher LVEF. Moreover, in obstructive HCM, these effects result in a reduction in LVOT obstruction, alleviation of symptoms and an improvement in exercise capacity [57]. A pre-specified subgroup analysis of the EXPLORER-HCM trial [57] showed that the effect of mavacamten on the primary composite endpoint of peak oxygen consumption (pVO2) and New York Heart Association (NYHA) class were greater in patients who were not taking BB during the study compared with those who were. This is largely explained by the blunting of heart rate response to exercise in treated patients. Moreover, in an echocardiographic substudy of the EXPLORER-HCM trial [58], mavacamten was shown to reduce parameters like systolic anterior motion (SAM) of the mitral valve, degree of mitral regurgitation, left atrial volume index, LV thickness, the ratio between mitral inflow velocity and annular early diastolic velocity (E/eʹ), annular early diastolic velocity (eʹ), and LVOT gradients. The main issue related to cardiac myosin inhibitors is the possibility of developing left ventricular systolic dysfunction, which is reversible with drug withdrawal and requires periodical echocardiographic monitoring [58]. The introduction of cardiac myosin inhibitors represents a promising advancement in the treatment of HCM, offering a targeted approach to address the underlying pathophysiology and to modify the abnormal PVR characteristic of this condition [59].

### 2.3 Cardiac Catheterization in HCM

Although Doppler echocardiography is often sufficient for the hemodynamic assessment of obstructive HCM, several conditions require invasive hemodynamic assessment.

#### 2.3.1 Evaluation of LVOT Obstruction

MR is a common feature of HCM which can contaminate the Doppler signal of LVOT obstruction. The severity of MR, whose jet in HCM is frequently eccentric, can be difficult to determine with echocardiography. In some patients, a high intracavitary velocity might be mistaken for an LVOT gradient [60]. For patients with multiple levels of obstruction (aortic valve stenosis, MVO, LVOT obstruction), the accurate quantitation of the severity of each lesion is best determined with cardiac catheterization [13]. For patients with symptoms out of proportion with the resting imaging studies, pharmacological provocation for dynamic LVOT obstruction in the cardiac catheterization laboratory should be considered [61, 62]. The dynamic nature of LVOT obstruction should be demonstrated with evidence of the “spike-and-dome” configuration in the aortic contour and further narrowing of the aortic pulse pressure on provocation (e.g., post-ectopic beat, Valsalva strain, amyl nitrate inhalation, or isoproterenol administration) [62].

#### 2.3.2 Evaluation of Transplant Candidates

Patients with end-stage HCM being considered for heart transplantation should undergo cardiac catheterization to assess their Interagency Registry for Mechanically Assisted Circulatory Support (INTERMACS) classification, pressures and resistances in the pulmonary circulation, and vasoreactivity of pulmonary hypertension, which can have prognostic implications when considering heart transplantation [63].

#### 2.3.3 Guidance During Invasive Procedures

Cardiac catheterization serves as a guide during invasive procedures such as septal alcoholization, providing real-time visualization of intracavitary pressure variations and outflow gradient changes [64].

#### 2.3.4 Hemodynamic Monitoring in Critical Care Settings

In patients with HCM and concomitant cardiogenic shock admitted to intensive care unit, bed-side cardiac catheterization allows for direct measurement of CO, filling pressures, pulmonary pressures and resistances, aiding in hemodynamic monitoring and therapeutical decision (e.g., titration of inotropic/vasopressor drugs, mechanical circulatory support) [65].

#### 2.3.5 Assess Exercise Response

An abnormal hemodynamic response to exercise is a notable feature in HCM. During exercise, the increase in LV filling pressures is multifactorial, involving impaired ventricular relaxation, increased stiffness, and external constraints. These elevated filling pressures contribute to left atrial enlargement and may eventually compromise atrial function, exacerbating dyspnea. Despite enlargement of the left atrium, its ability to boost pump function remains intact, as evidenced by consistently high “a” waves in PCWP tracings at rest and during peak exercise. This highlights the necessity of elevated filling pressures in patients with significant diastolic chamber stiffness. These elevated pressures, reflected back into pulmonary venous circulation, are likely key contributors to dyspnea [42].

## 3. Dilated Cardiomyopathy (DCM)

DCM is characterized by ventricular dilation and systolic dysfunction, occurring in the absence of severe coronary artery disease, valvular disease, hypertension, congenital heart disease or any other abnormal loading condition as a primary cause of cardiac dysfunction [66]. At transthoracic echocardiography LV dilation in adults is defined as a left ventricular end-diastolic diameter (LVEDD) >58 mm in males and >52 in females and a LVEDV index of ≥75 mL/m^2^ in males and ≥62 mL/m^2^ in females [1]. DCM is one of the most common causes of HF and it has an estimated prevalence of 1:250 individuals. This cardiomyopathy results from a variety of causes that can be genetic or acquired (i.e., drugs and toxins, infectious agents, and endocrine disturbances) although many cases are idiopathic [66]. The main pathophysiological feature of DCM is ventricular systolic dysfunction, defined by LVEF <50% [1]. The reduced contractility of sarcomeres leads to an expansion in ventricular volumes as a compensatory mechanism to uphold CO through the Frank-Starling law. Although elevated preload initially enhances ventricular contractility, this pressure and volume overload ultimately leads to a plateau and subsequent decline in myocardial contraction. This process results in cardiac remodeling characterized by thin-walled dilated ventricles [67].

### 3.1 PVR in DCM

**Fig. 2.**
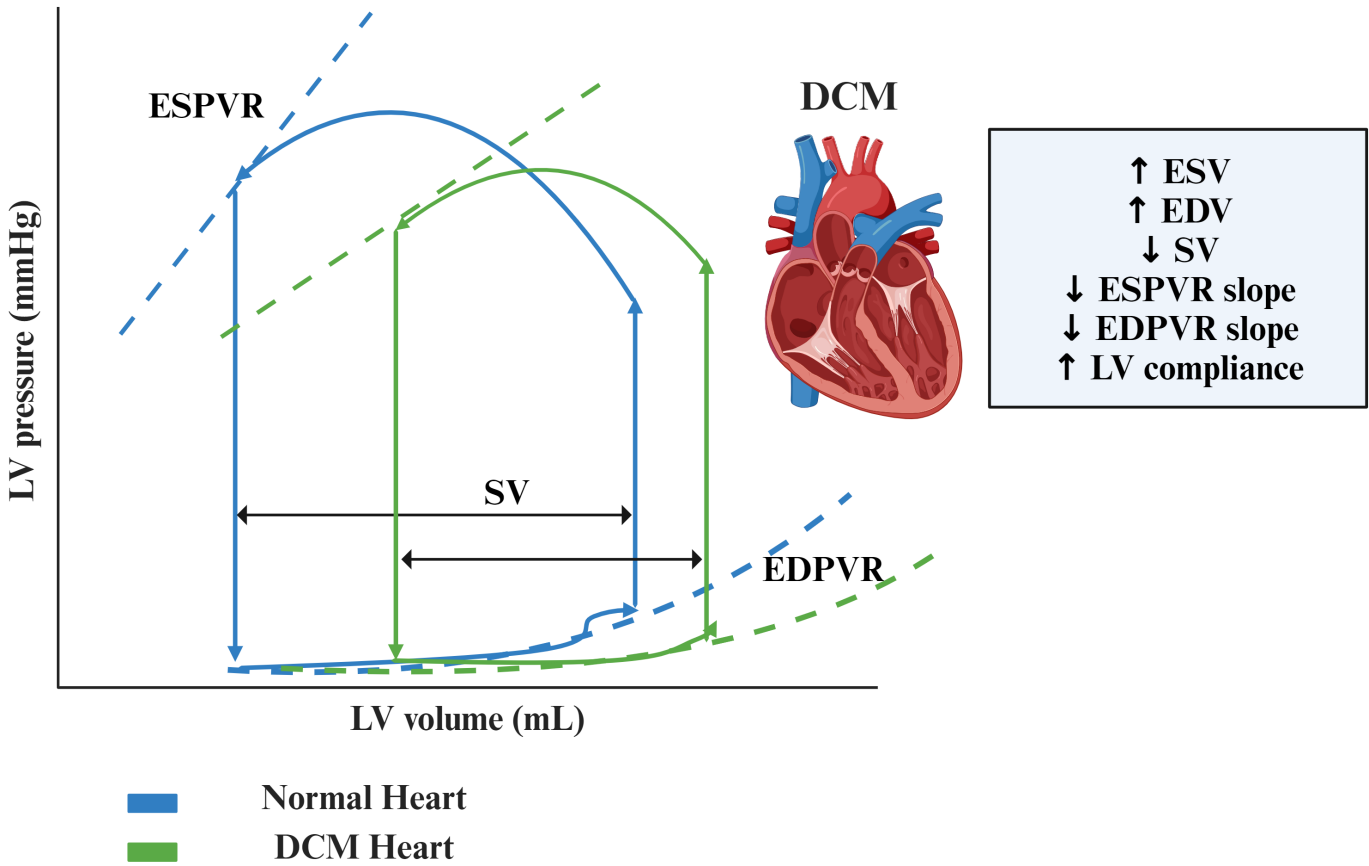
**Pressure-volume loops in DCM in initial phases compared to normal heart**. Abbreviations: DCM, dilated cardiomyopathy; EDPVR, end-diastolic pressure-volume relationship; EDV, end-diastolic volume; ESPVR, end-systolic pressure-volume relationship; ESV, end-systolic volume; LV, left ventricle; SV, stroke volume. ↑ increase, ↓ decrease.

The impaired mechanics of the heart due to its enlarged and weakened state alters the left ventricular contraction and relaxation. As for HCM, in patients with DCM PVR may exhibit various anomalies compared to healthy subjects (Fig. 2).

#### 3.1.1 Increase in ESV and EDV

As a response to volume overload and myocyte loss, DCM hearts undergo eccentric remodeling, resulting in an enlarged LV cavity with a thin myocardial wall, with a subsequent increase in EDV and ESV [68]. The increase in LV volumes may be aggravated by the development of secondary MR, which causes further volume overload and thus LV dilation and is associated with a worse prognosis [69].

#### 3.1.2 Decreased Slope and Rightward Shift of ESPVR

This reflects both the increase in LVEDV and left ventricular end-systolic volume (LVESV), which determines a stretching of the myocardium, and a reduced intrinsic contractility resulting in a decrease of Ees. The reduction in intrinsic myocardial contractility is secondary to three main mechanisms: the dysfunction of contractile proteins, the alteration of excitation-contraction coupling and an impairment of myocardial relaxation [70].

#### 3.1.3 Reduced SV and CO

The reduced LV contractility leads to a decrease in left ventricular end-systolic pressure (LVESP) and SV, despite the augmented preload. As Ees decreases, LVESP, SV and LVEF tend to decrease as well, whereas LVEDP and heart rate increase. Indeed, given the ventricle weakening, the heart tries to pump faster to increase CO [71]. Once the preload reserve is depleted, SV begins to be influenced by variations in the afterload which is determined by several factors such as vascular distensibility, vascular resistance, blood viscosity, and primarily myocardial wall tension. Ventricular dilation directly causes an increase in wall stress and oxygen consumption, resulting in augmented afterload and a consequent reduction in SV, unless a sufficient degree of compensatory eccentric myocyte hypertrophy is established to normalize wall tension [70, 71]. However, as the disease progresses and HF occurs, wall stress becomes too elevated and the afterload progressively increases, leading to a further decline in SV [72, 73].

#### 3.1.4 Alterations in EDPVR

In the initial phases of the disease, LV filling pressures are relatively low. This is represented in PVR by a rightward and downward shift in EDPVR with a slight reduction in EDPVR slope, reflecting a mild increase in compliance. However, in the advanced phases of the disease, the greater fibrotic burden which causes increased LV stiffness, the augmentation of filling pressures and the reduction in SV and CO lead to diastolic dysfunction with an upward and rightward shift of PVR, an increase of EDPVR slope and therefore a reduction in LV compliance [74].

### 3.2 Pharmacological Therapy of DCM and Changes in PV Loops 

Treatment strategies in DCM reflect the management of chronic HF. BB, renin-angiotensin-aldosterone system (RAAS) inhibitors and, more recently, sodium-glucose cotransporter 2 inhibitors (SGLT2-I) are considered the cornerstones of DCM therapy [75]. These agents play an important role in the “reverse remodeling” phenomenon by reducing LV volumes with an improvement in systolic function [76]. The hemodynamic effects of drugs currently used for DCM management are displayed in Table 2.

**Table 2.** **Hemodynamic effects of pharmacological therapy in DCM**.

	BB	RAAS inhibitors	SGLT2-I	Cardiac myosin activators
LVEDV	↓	↓	↓	↓
LVESV	↓↓	↓↓	↓	↓
SV	↑	↓	↑	↑
LVEDP	↓	↓	↓	↔/↑
ESPVR slope	↑	↑	↑	↑
EDPVR slope	↓	↓	↓	↔/↑
LV fibrosis	↔	↓	↓	↓

Abbreviations: BB, beta-blockers; DCM, dilated cardiomyopathy; EDPVR, end-diastolic pressure-volume relationship; ESPVR, end-systolic pressure-volume relationship; LV, left ventricular; LVEDP, left ventricular end-diastolic pressure; LVEDV, left ventricular end-diastolic volume; LVESV, left ventricular end-systolic volume; RAAS, renin-angiotensin-aldosterone system; SGLT2-I, sodium-glucose cotransporter 2 inhibitors; SV, stroke volume. ↑ increase, ↓ decrease, ↔ no change.

#### 3.2.1 BB

Congestive HF is associated with sustained activation of the sympathetic nervous system which leads to a reduction in myocardial β-adrenergic receptor density and an impaired contractile response to catecholamine stimulation [77]. BB in DCM confer a benefit in LV function, including improvement in NYHA functional class and mean exercise capacity, with a favorable impact on patient survival [78]. In symptomatic patients with moderate-to-severe cardiac dysfunction, long-term administration of metoprolol was associated with an increase in myocardial β-receptor density leading to a significant improvement in resting hemodynamic output (stroke work index and [77] LVEF) and in contractile response to catecholamine stimulation, with an increase in peak positive LV contractility after dobutamine infusion [77]. BB can also improve LV diastolic properties leading to more complete myocardial relaxation which ultimately results into a decrease in LVEDP [79]. Among BB, carvedilol appears to be the most effective in improving cardiac function, ventricular remodeling and clinical efficacy in patients with DCM [80].

#### 3.2.2 RAAS Inhibitors

Angiotensin converting enzyme inhibitors (ACE-I) and angiotensin II receptor blockers (ARBs) effectively block the RAAS and delay the progression of HF and myocardial fibrosis in DCM. These drugs allow a decrease in the LVEDV and LVESV accompanied by a leftward shift in the diastolic pressure-volume loop and an improvement in LV mass and sphericity indexes. Furthermore, ACE-I improve transmural myocardial perfusion and restore impaired subendocardial coronary flow and vasodilator reserve in DCM though bradykinin-mediated and nitric oxide-dependent mechanisms. The beneficial effect of these agents results in a reduction in HF hospitalizations and death rates [81, 82, 83, 84]. Mineralocorticoid receptor antagonists (MRA), in addition to standard therapy for HF, are also effective in improving symptoms and in reducing the risk of cardiovascular death among patients with severe LV systolic dysfunction [85]. By preventing the sympathetic activation and the profibrotic effect mediated by aldosterone, these drugs are useful for improving ventricular remodeling with a reduction in ventricular volumes and stiffness in association with a regression of myocardial fibrosis. This results in amelioration of systolic and diastolic ventricular function in symptomatic patients [86, 87].

A beneficial effect on ventricular reverse remodeling has also been observed with the use of sacubitril/valsartan, an agent that belongs to the class of angiotensin receptor-neprilysin inhibitors (ARNI) and acts on both the RAAS and the neutral endopeptidase systems [88]. This drug has been shown to improve ventricular function with a concomitant decrease in ventricular dimensions in patients with non-ischemic DCM, resulting in lower rates of hospitalization and cardiac mortality, as well as improved functional status [89, 90].

#### 3.2.3 SGLT2-I

SGLT2-I inhibit renal glucose reabsorption, thereby increasing urinary glucose excretion and reducing the glucose load in the body. These agents exhibit pleiotropic effects, the mechanisms of which are not yet fully elucidated, resulting in a reduction in inflammation and in sympathetic overdrive [91]. Treating HF patients with SGLT2-I has resulted in a lower rate of hospitalization and cardiovascular death [92]. Hong *et al*. [93] found that at 12-month follow-up, patients with DCM receiving dapagliflozin in addition to conventional medical therapy had a greater absolute percentage fall in LV volumes with a more significant improvement in cardiac function, compared to patients undergoing conventional therapy only. These results suggest a possible role of SGLT2-I in promoting LV reverse remodeling.

#### 3.2.4 Cardiac Myosin Activators

Cardiac myosin activators are a new class of drugs which have been shown to improve myocardial contractility increasing LV systolic pressure generation per unit time (dP/dt), therefore increasing hemodynamic parameters, including CO and SV. A remarkable feature of these drugs is that they augment myocardial performance in a calcium-independent manner, conversely from traditional inotropes [94].

Omecamtiv mecarbil is an allosteric modulator which directly activates cardiac myosin in a calcium-independent manner, increasing the number of myosin heads that are able to bind to actin filaments during depolarization and the total amount of time spent in contraction and systole, without increasing intracellular calcium [95]. The main hemodynamic effects of omecamtiv mecarbil have been clarified in a phase-2 study (COSMIC-HF) conducted in patients with heart failure with reduced ejection fraction (HFrEF). This new drug has been shown, after 20 weeks of administration, to increase LV systolic ejection time and SV, to decrease LVEDV and LVESV (suggesting a potential role in reverse remodeling) and to reduce HR and natriuretic peptide levels [96]. However, omecamtiv mecarbil prolongs the duration of systole at expense of diastolic time. This reflects in a leftward shift of PVR and a potential increase in EDPVR slope and LV filling pressures, which may be harmful especially at elevated HR [97]. GALACTIC-HF is a phase 3 placebo-controlled RCT which studied the effects of omecamtiv mecarbil in a cohort of patients with symptomatic chronic HFrEF in addition to conventional therapy. The primary outcome of reduction of a composite of HF events and cardiovascular death was reached, with a slightly lower incidence in the omecamtiv mecarbil group. In this study, omecamtiv mecarbil was shown to be safe as it did not increase myocardial ischemia, ventricular arrhythmias and cardiac death. This could be explained by the fact that, unlike other inotropes, this drug acts in a calcium-independent manner [98].

### 3.3 Cardiac Catheterization in DCM

Hemodynamic assessment through cardiac catheterization in DCM should be reserved for specific scenarios, i.e., in patients with advanced disease eligible for cardiac transplantation, as its diagnostic value is limited. Nonetheless, it remains crucial for the prognostic assessment. Compensated HF in DCM manifests with elevated LVEDP, alongside a relatively normal diastolic pressure prior to atrial contraction and a normal mean PCWP at rest. Indeed, impaired myocardium relaxation causes a low-pressure gradient between the left atrium and the left ventricle during early diastole, leading to reduced early ventricular filling. However, in late diastole, following atrial contraction, ventricular filling increases resulting in elevated LVEDP [99, 100]. Moreover, left atrial pressure generally rises only during the latter part of the diastolic phase. Therefore, mean left atrial pressure (corresponding to mean PCWP) is not significantly elevated [101]. Conversely, in decompensated HF left ventricular pressure is increased throughout diastole because the ventricular early diastolic compliance is overwhelmed. Indeed, volume overload of the left atrium causes an increase in the left atrial pressure throughout the whole cardiac cycle, resulting in a high atrio-ventricular gradient even during early diastole and, consequently, in elevated early ventricular filling. Furthermore, mean PCWP increases as well, equalizing to LVEDP, or getting even higher if atrial compliance is particularly altered [18, 102]. However, even in compensated DCM there may be a high left atrial pressure during early diastole, especially in cases of severe MR. Indeed, excessive ventricular dilation can lead to severe functional MR due to LV enlargement and annular dilation. This condition results in a high early atrio-ventricular pressure gradient, with normal mean PCWP and elevated LVEDP [103]. Although compensated patients have a normal mean PCWP at rest, during exercise or tachycardia they may have a rise in left atrial pressure and an inadequate increase in SV, resulting in exertional dyspnea and fatigue. Moreover, in the early stages of the disease, the diastolic pulmonary artery pressure tends to be equal to PCWP. However, as the disease progresses, patients may develop severe PH. Indeed, chronic HF and persistently elevated PCWP (>25 mmHg) over time result in passive postcapillary PH and in precapillary PH due to arterial remodeling with increased pulmonary vascular resistance [104].

#### Hemodynamic Findings Related to Long-Term Prognosis

Although DCM prognosis has improved in recent years, LV dysfunction associated with signs of congestive HF is still characterized by a dismal prognosis, especially in those patients with a “wet” or “cold” profile [105]. High filling pressure, especially when combined with low CO, and increased PCWP and pulmonary vascular resistance are some hemodynamic indexes suggesting a worse course of the disease [74, 106]. In particular, the persistence of restrictive filling pattern three months post-presentation correlates with high mortality and transplantation rates. Conversely, patients with reversible restrictive filling exhibit excellent prognosis due to improved ventricular reverse remodeling [107]. Furthermore, moderate to severe functional MR at diagnosis or the persistent severe regurgitation despite optimal medical treatment correlate with a poor prognosis and an increased need for invasive therapeutic strategies [108]. Finally, recent noninvasive techniques for evaluating myocardial deformation, such as speckle-tracking echocardiography, demonstrate higher sensitivity than LVEF in detecting subclinical abnormalities of systolic function [109]. Global longitudinal strain seems to correlate with invasive hemodynamic parameters and its worsening is associated with an increased risk of long-term adverse cardiac events, thus it might be used to identify high-risk patients [110].

## 4. Restrictive Cardiomyopathy (RCM)

RCM is often regarded as the rarest form of myocardial disease, representing 2% to 5% of cases [111], posing considerable challenges in its definition and classification. It encompasses a range of disorders with unique difficulties in classification and diagnosis. RCM presents the widest array of causes and histological characteristics among cardiomyopathies, often necessitating cardiac catheterization or endomyocardial biopsy (EMB) for definitive diagnosis [111]. Adding to the complexity, the boundaries of RCM are increasingly blurred due to shared disease-causing genes with other cardiomyopathies [112] and potential changes in cardiac phenotypes over time. While the hemodynamic definition of restrictive physiology is clear, there can be variations in PVR, and diagnostic cutoffs for restriction are not straightforward. Additionally, advanced imaging modalities such as cardiovascular magnetic resonance (CMR), bone tracer scintigraphy and positron emission tomography aid in identifying specific causes of tissue damage (e.g., amyloidosis, Fabry disease, hemochromatosis, sarcoidosis), even before typical restrictive physiology fully manifests [112].

In its primary form, RCM is usually inherited in an autosomal dominant manner, while less frequently it presents as autosomal recessive or sporadic. The most common gene associated with RCM is *TNNI3*, which encodes the troponin I, a thin filament protein [113]. Other genes which may cause primary RCM belong to cytoskeleton, sarcomeric proteins and regulatory proteins. Less frequently, accumulation in the intracellular space of defective or misfolded proteins, like desmin and filamin-c, may cause a restrictive phenotype as well [114].

According to the current ESC guidelines on cardiomyopathies [1], RCM is defined by the presence of restrictive physiology, often accompanied by atrial enlargement and non-dilated ventricles, irrespective of ventricular wall thickness and systolic function. However, the ESC definition does not take into account the histological substrate, which is crucial in the RCM definition. Indeed, although histological alteration may involve the myocardium (in the intracellular or the interstitial space) or the endocardium, it is possible that forms of RCM lack the gross abnormalities as the underlying primary pathophysiological alteration may be determined by cellular function [112].

Patients affected by RCM exhibit a stiff LV with compromised diastolic filling and elevated filling pressures. Prolonged elevation of LV diastolic pressures frequently induces PH, which can worsen right ventricular dysfunction, particularly in cases where the RV is also affected, as observed in cardiac amyloidosis (CA). In the initial stages of RCM, LV systolic function typically remains intact, at least as assessed by LVEF, but tends to degrade with time. Longitudinal LV systolic function is often diminished in the early phases, especially in CA. Despite preserved LVEF, the LV struggles to fill adequately, and ventricular chamber size may decrease in the presence of markedly increased wall thickness, resulting in nearly fixed SV. In such circumstances, the sole adaptive response to exercise capable of augmenting CO is an increase in heart rate, which might be attenuated in patients with concurrent autonomic dysfunction, heightening the risk of hypotension during exertion. Additionally, remodeling and dilation of the atria frequently lead to AF, diminishing atrial contribution to LV filling [115].

### 4.1 PVR in RCM 

**Fig. 3.**
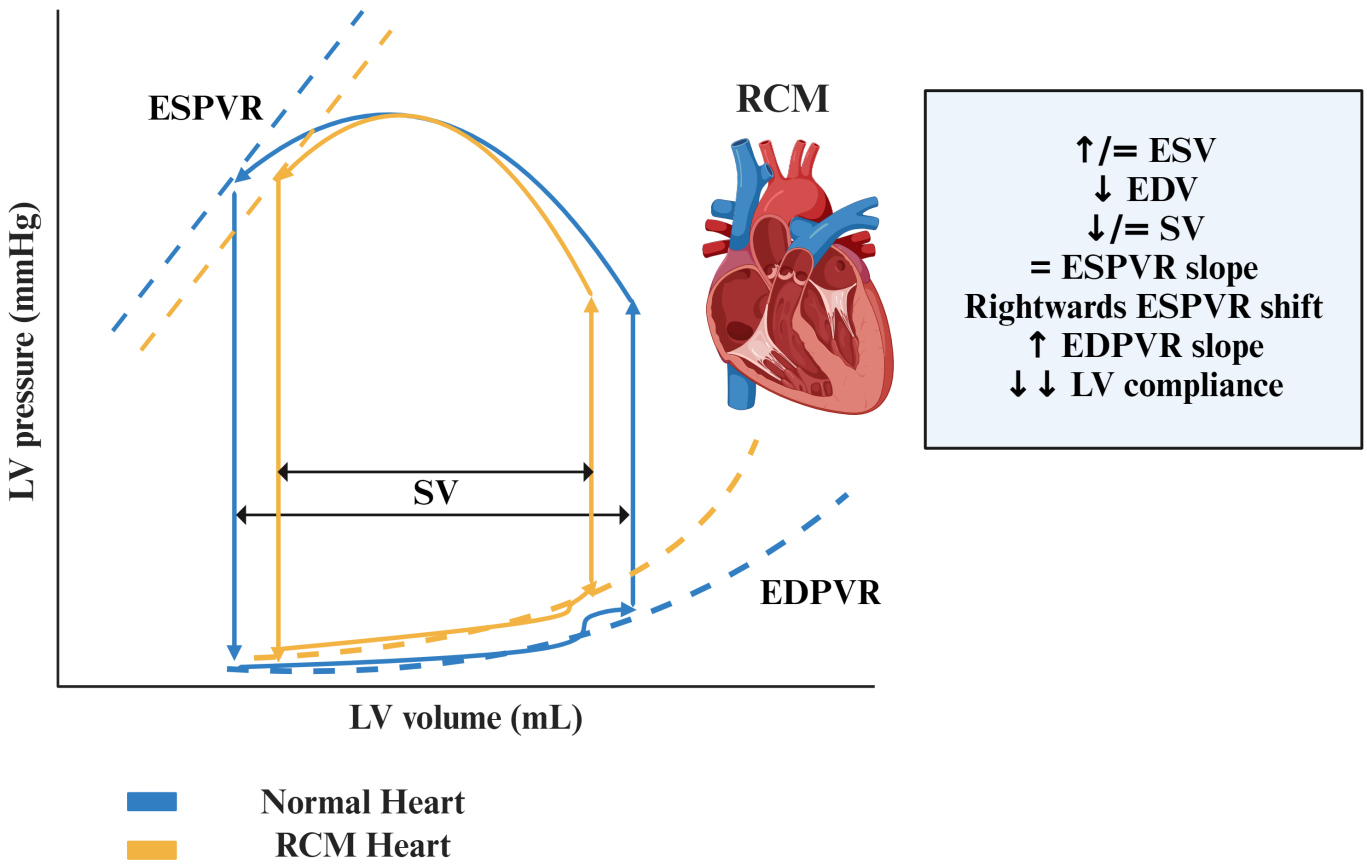
**Pressure-Volume loops in RCM compared to normal hearts**. Abbreviations: EDPVR, end-diastolic pressure-volume relationship; EDV, end-diastolic volume; ESPVR, end-systolic pressure-volume relationship; ESV, end-systolic volume; LV, left ventricle; RCM, restrictive cardiomyopathy; SV, stroke volume. ↑ increase, ↓ decrease, = no change.

Just as in HCM and DCM, hemodynamic alterations are also present in RCM, which cause the PVR to differ from that of normal subjects. The main features of PVR in RCM are displayed in Fig. 3.

#### 4.1.1 Preserved Left Ventricular Contractility – ESPVR Slope Unchanged

RCM is one of the main models of heart failure with preserved ejection fraction (HFpEF). As occurs in HFpEF, LVEF is preserved, occasionally showing mild signs of contractile dysfunction. In RCM, ESPVR is usually shifted rightwards (due to small LV volumes) and shows an unchanged or mildly increased slope, which represents Ees. This is usually accompanied by a proportional increase of effective arterial elastance (Ea), therefore the ratio Ea/Ees, which represents ventriculo-arterial coupling, remains stable [3].

#### 4.1.2 Increased EDPVR Slope

In RCM the main hemodynamic feature is an overt diastolic dysfunction, which is mainly caused by increased myocardial stiffness. In PVR this is represented by a marked increase in the slope of EDPVR and therefore in a reduction in LV compliance.

#### 4.1.3 Reduction in EDV

A left ventricle with restrictive physiology, together with an increased slope of EDPVR and therefore a decreased compliance, will exhibit a smaller EDV due to impaired filling at a given pressure. ESV is usually unchanged or mildly increased leading to a reduction in SV. CO, however, is maintained thanks to a proportional increase in heart rate.

#### 4.1.4 Increase of LVEDP and PCWP

In RCM hearts, due to chamber stiffness, LVEDP is higher than normal hearts: during exercise, PCWP increases proportionally to the increase in pulmonary wedge associated with severe symptoms of dyspnea [65].

### 4.2 Pharmacological Therapy of RCM and Changes in PV Loops

To date, no pharmacological agents have been definitively proven to alter the natural progression of most forms of RCM. This underscores a significant gap in the current management of the disease, where treatment is primarily focused on symptomatic relief and managing complications such as heart failure and arrhythmias.

In certain subtypes of RCM, such as CA and Fabry disease (FD), therapeutical agents which target the molecular mechanism of the disease have been studied and shown to change the natural course of the disease.

CA is caused by the accumulation of insoluble protein fibrils in the extracellular space. The two main subtypes are light chain (AL-CA) and transthyretin (ATTR-CA). AL-CA is due to the overproduction of antibody light chains, which misfold and deposit in tissues, while ATTR-CA arises from the extracellular buildup of misfolded transthyretin (TTR) monomers and can be classified in a wild-type form or a hereditary one [116]. Treatment of AL-CA is based on reduction of light chain production, which can be achieved with chemotherapy, followed by autologous stem cell transplantation in eligible patients. More recently, antifibril antibodies have been studied in phase 3 randomized controlled trials (RCTs) and have been shown promising results in treating this condition [117]. With regard to ATTR-CA, the currently available drugs can be divided in two main classes: stabilizers of TTR, which act by inhibiting the dissociation of TTR in monomers and therefore the precipitations of amyloid in tissues and include tafamidis and acoramidis [118, 119]; and drugs inhibiting hepatic TTR production (small-interfering RNA, as patisiran and vutisiran, and antisense oligonucleotide, as inotersen and eplontersen) [120, 121, 122, 123]. More recently, gene-therapy based on degradation of TTR by the CRISPR-Cas9 system has been studied as an additional therapeutical agent [124].

FD is a recessive X-linked multisystem lysosomal storage disorder caused by a mutation of alpha-galactosidase A gene (*GLA*) resulting in accumulation of globotriaosylceramide (GL3) in various organs (heart, kidney, nervous system) [125]. Currently available drugs for treatment of FD are represented by enzyme-replacement therapy (ERT) and chaperone therapy, which can only be used in selected mutations and in patients with residual enzymatic activity [126, 127]. Starting these drugs early, before the development of myocardial fibrosis, is crucial to achieve greater benefit and long-term improvement of cardiac function [128]. Finally, as for CA, gene therapy, either with viral vector or gene-editing systems, is under evaluation in clinical studies for FD [129].

The main effects of drugs on hemodynamics and PVR of RCM are displayed in Table 3.

**Table 3.** **Hemodynamic effects of pharmacological therapy in RCM**.

	BB/CCB	Diuretics	SGLT2-I
LVEDV	↓	↓	↓
HR	↓	↔	↔
SV	↑	↔/↓	↔
ESPVR slope	↓	↔	↔/↑
LVEDP	↓	↓	↓
LV fibrosis	↔	↔	↓

Abbreviations: BB, beta-blockers; CCB, calcium-channel blockers; ESPVR, end-systolic pressure-volume relationship; HR, heart rate; LV, left ventricular; LVEDP, left ventricular end-diastolic pressure; LVEDV, left ventricular end-diastolic volume; RCM, restrictive cardiomyopathy; SGLT2-I, sodium-glucose cotransporter 2 inhibitors; SV, stroke volume. ↑ increase, ↓ decrease, ↔ no change.

#### 4.2.1 BB and CCB

These two classes of drugs have been widely used in RCM mainly for their hemodynamic impact on heart rate and myocardial relaxation. However, in specific forms of RCM (such as CA) these drugs have some limitations and detrimental effects. In fact, both BB and CCB may be poorly tolerated in CA patients due to adverse reactions as hypotension, bradycardia and poor effort tolerance. All these effects may be more pronounced in CA because of concomitant peripheral and autonomic neuropathy. Moreover, severe bradycardia may require a pacemaker implantation due to strict dependance of CO on HR [130].

The main hemodynamic effects and the impact on PVR of BB and CCB in RCM are summarized as follows.

4.2.1.1 Reduced Heart Rate and an Increase in LVEDV and SV

By slowing heart rate, BB extend diastole, potentially improving ventricular filling time. This may lead to a slight increase in EDV and SV, despite the underlying stiffness.

4.2.1.2 Decreased Myocardial Oxygen Demand

Lowering heart rate and contractility reduces myocardial oxygen consumption, which can be beneficial in a condition where myocardial perfusion may be compromised due to high filling pressures. However, since the length of diastole and the elevated filling pressure have a relatively minor effect on the volume of ventricular filling, resulting in a nearly fixed SV, CO is critically reliant on changes in heart rate. Indeed, BB and CCB could be detrimental and not be tolerated due to their negative chronotropic and, to a lesser extent, inotropic impact (decreasing the Ees), without improving SV [131].

4.2.1.3 Rhythm Control

AF is a common feature of RCM and often poorly tolerated because of the loss of atrial contribution to ventricular filling. Therefore, restoring sinus rhythm results in an improvement of SV. Consequently, rhythm control should be prioritized over rate control, even though achieving and sustaining sinus rhythm can be challenging [132].

#### 4.2.2 Diuretics

Diuretics are traditionally considered a cornerstone therapy in management of HFpEF and therefore in RCM due to their action in managing fluid overload and treating congestive symptoms [133].

The main limitation of diuretic therapy in RCM is that, if not adequately prescribed or titrated, they can cause an excessive reduction in LVEDV and therefore in SV. Hence, forcing diuresis should be avoided because even mild hypovolemia may result in hemodynamic deterioration with a dramatic reduction of CO [112].

4.2.2.1 Reduction in LVEDV

Diuretics decrease intravascular volume, which lowers the filling pressures (preload) in the heart. This is particularly beneficial in RCM where elevated ventricular filling pressures are a primary issue.

4.2.2.2 Decrease in LVEDP

The diastolic portion of the loop moves downward, showing a reduction in LVEDV. This change is due to lower filling pressures following volume reduction.

4.2.2.3 Unchanged or Slightly Reduced SV

Since RCM is characterized by impaired ventricular filling, SV may remain relatively unchanged or slightly reduced due to the reduced EDV. However, the overall hemodynamic status improves due to lower congestion resulting in decreasing symptoms [134].

#### 4.2.3 SGLT2-I

These new pharmacological agents represent the first class of drugs that have shown a significant prognostic benefit in HFpEF in clinical trials. Key clinical trials such as EMPEROR-Preserved [135] and DELIVER [136] have demonstrated that these drugs can reduce the risk of HF hospitalization in patients with HFpEF. Given the pathophysiological similarities between HFpEF and RCM, particularly in terms of diastolic dysfunction and elevated filling pressures, SGLT2-I might offer similar benefits in RCM. However, it is important to note that direct evidence from clinical trials specifically investigating the effects of SGLT2-I in RCM is still limited.

4.2.3.1 Reduction in Preload and Afterload

SGLT2-I promote natriuresis and diuresis, leading to a decrease in plasma volume and blood pressure. This reduces both preload and afterload, which can be particularly beneficial in conditions like RCM where elevated filling pressures are a primary concern [137].

4.2.3.2 Improved Myocardial Energetics

SGLT2-I may enhance myocardial energy metabolism by promoting a shift from glucose to ketone utilization, which is a more efficient energy substrate for the heart. This metabolic shift could potentially improve cardiac function in RCM [138].

4.2.3.3 Reduction of Interstitial Fibrosis

One animal study suggests that SGLT2-I may reduce myocardial fibrosis, a common feature in RCM, thereby improving myocardial compliance and diastolic function [139].

### 4.3 Cardiac Catheterization in RCM

Through invasive hemodynamic evaluation, RCM is distinguished by elevated diastolic filling pressures and a rapid equalization of filling pressures among the four cardiac chambers during diastole, often displaying a frequent ‘dip and plateau’ or ‘square root’ pattern on pressure tracings [112]. This pattern becomes more conspicuous with maneuvers that enhance ventricular filling, such as volume infusion or leg raise. Although many of these observations overlap with constrictive pericarditis (CP), several distinctions exist between these two conditions. For instance, atrial x and y descents are typically somewhat subdued in RCM compared to CP, and a wave may be attenuated when the atria are primarily affected (such as in CA). Disproportionate stiffness in the left heart may lead to moderate pulmonary hypertension, which is less common and less severe in CP. Additionally, the RCM process renders the chambers minimally compliant; hence, there is minimal respiratory variation in flow or pressure, unlike CP. Ventricular interdependence is minimal in RCM, resulting in little alteration in peak ventricular systolic pressures with respiration [140, 141]. The main differences with CP are shown in Table 4.

**Table 4.** **Difference between RCM and CP at cardiac catheterization**.

	RCM	CP
Ventricular filling	Early rapid ventricular filling due to high atrial pressures, followed by limitation in filling from stiff myocardium.	Accentuated early rapid ventricular filling followed by a sudden rapid rise in pressure due to pericardial restraint.
Pericardial/Myocardial compliance	Stiff, noncompliant ventricles unable to easily accept additional increments in volume during atrial contraction.	Reduced pericardial compliance limits myocardial stretch during diastole.
Pressures variation with respiration	No significant variation of systolic pressures with respiration and concordance of RV and LV pressure.	Rise in RV peak pressure and fall in LV peak pressure during inspiration (with the opposite during expiration). Discordance of systolic pressures with respiration due to ventricular interdependence.
Kussmaul’s sign	May be present, but less specific compared to CP.	Present, with increased jugular venous pressure during inspiration.
Jugular venous pressure	Prominent “y” descent, but no pronounced “x” descent. A wave may be attenuated due to atrial disease or absent (AF).	Elevated jugular venous pressure with prominent “x” and “y” descents.

Abbreviations: AF, atrial fibrillation; CP, constrictive pericarditis; LV, left ventricle; RCM, restrictive cardiomyopathy; RV, right ventricle.

### 4.4 Echocardiographic Findings in RCM 

The hemodynamic profile shared by all variants of RCM can be accurately delineated through a transthoracic echocardiogram. The initial indication of restrictive physiology manifests as concurrent enlargement of both atria (unrelated to specific causes like valve disorders or AF), LV and right ventricle ejection fraction within normal limits or mildly reduced, and non-dilated ventricles. Doppler imaging subsequently reveals a restrictive filling pattern of transmitral flow, characterized by heightened early diastolic filling velocity (E wave) owing to elevated left atrial pressure, and diminished atrial filling velocity (A wave) due to elevated ventricular diastolic pressure, shortening mitral deceleration time and IVRT. Moreover, the ratio of systolic to diastolic pulmonary venous flow markedly diminishes due to elevated left atrial pressures. Tissue Doppler typically indicates decreased e^′^, resulting in an elevated E/e^′^ ratio. Congestion of the inferior vena cava and hepatic veins, along with diastolic flow reversal in the hepatic veins during inspiration, are frequently observed, reflecting the inability of a non-compliant RV to accommodate increased venous return [142]. The main echocardiographic differences between RCM and CP are shown in Table 5.

**Table 5.** **Echocardiographic differences between RCM and CP**.

	RCM	CP
Transmitral flow	No respiratory E variation	Respiratory E variation ≥25%
	DT <150 msec	DT generally >150 msec
Transtricuspid flow	Respiratory E variation ≤15%	Respiratory E variation ≥35%
Mitral annulus TDI	E ≤8 cm/sec	E >8 cm/sec
E/e’ ratio	Increased (≥15)	Normal (<15)
Hepatic vein flow	↑ Inspiratory flow reversal	↑ Expiratory
Interventricular septum	Usually not affected	Paradoxical motion (“septal shift” and “septal bounce”)
Pericardium	Usually not affected	Thickened/calcifications

Abbreviations: CP, constrictive pericarditis; DT, deceleration time; RCM, restrictive cardiomyopathy; TDI, tissue doppler imaging.

## 5. Conclusions

Cardiomyopathies, due to increased attention and advancements in imaging diagnostic techniques, are no longer considered rare conditions. Understanding their hemodynamic aspects and the expected changes with the use of certain drugs is crucial for managing patients in both chronic and acute settings. Additionally, the evolution from one phenotype to another is not uncommon. The use of pressure-volume curves in clinical practice is quite limited, but non-invasive estimation techniques could potentially aid actual clinical practice.

## References

[1] Arbelo E, Protonotarios A, Gimeno JR, Arbustini E, Arbelo E, Barriales-Villa R (2023). 2023 ESC Guidelines for the management of cardiomyopathies: Developed by the task force on the management of cardiomyopathies of the European Society of Cardiology (ESC). *European Heart Journal*.

[2] Elliott P, Andersson B, Arbustini E, Bilinska Z, Cecchi F, Charron P (2008). Classification of the cardiomyopathies: a position statement from the European Society Of Cardiology Working Group on Myocardial and Pericardial Diseases. *European Heart Journal*.

[3] Protti I, van den Enden A, Van Mieghem NM, Meuwese CL, Meani P (2024). Looking Back, Going Forward: Understanding Cardiac Pathophysiology from Pressure-Volume Loops. *Biology*.

[4] Sandoval Y, Burke MN, Lobo AS, Lips DL, Seto AH, Chavez I (2017). Contemporary Arterial Access in the Cardiac Catheterization Laboratory. *JACC. Cardiovascular Interventions*.

[5] Seferović PM, Polovina M, Rosano G, Bozkurt B, Metra M, Heymans S (2023). State-of-the-art document on optimal contemporary management of cardiomyopathies. *European Journal of Heart Failure*.

[6] Connors AF, Speroff T, Dawson NV, Thomas C, Harrell FE, Wagner D (1996). The effectiveness of right heart catheterization in the initial care of critically ill patients. SUPPORT Investigators. *JAMA*.

[7] Bastos MB, Burkhoff D, Maly J, Daemen J, den Uil CA, Ameloot K (2020). Invasive left ventricle pressure-volume analysis: overview and practical clinical implications. *European Heart Journal*.

[8] Writing Committee Members, Ommen SR, Ho CY, Asif IM, Balaji S, Burke MA (2024). 2024 AHA/ACC/AMSSM/HRS/PACES/SCMR Guideline for the Management of Hypertrophic Cardiomyopathy: A Report of the American Heart Association/American College of Cardiology Joint Committee on Clinical Practice Guidelines. *Journal of the American College of Cardiology*.

[9] Maron BJ (2018). Clinical Course and Management of Hypertrophic Cardiomyopathy. *The New England Journal of Medicine*.

[10] Maron BJ, Rowin EJ, Maron MS (2022). Hypertrophic Cardiomyopathy: New Concepts and Therapies. *Annual Review of Medicine*.

[11] Maron BJ, Maron MS (2013). Hypertrophic cardiomyopathy. *Lancet (London, England)*.

[12] Maron BJ, Desai MY, Nishimura RA, Spirito P, Rakowski H, Towbin JA (2022). Diagnosis and Evaluation of Hypertrophic Cardiomyopathy: JACC State-of-the-Art Review. *Journal of the American College of Cardiology*.

[13] Geske JB, Sorajja P, Ommen SR, Nishimura RA (2011). Variability of left ventricular outflow tract gradient during cardiac catheterization in patients with hypertrophic cardiomyopathy. *JACC. Cardiovascular Interventions*.

[14] Losi MA, Betocchi S, Aversa M, Lombardi R, Miranda M, Cacace A (2003). Dobutamine stress echocardiography in hypertrophic cardiomyopathy. *Cardiology*.

[15] Gotsman MS, Lewis BS (1974). Left ventricular volumes and compliance in hypertrophic cardiomyopathy. *Chest*.

[16] Popescu BA, Rosca M, Schwammenthal E (2015). Dynamic obstruction in hypertrophic cardiomyopathy. *Current Opinion in Cardiology*.

[17] Murgo JP, Alter BR, Dorethy JF, Altobelli SA, McGranahan GM (1980). Dynamics of left ventricular ejection in obstructive and nonobstructive hypertrophic cardiomyopathy. *The Journal of Clinical Investigation*.

[18] Braunwald E, Brockenbrough EC, Frahm CJ, Ross J (1961). Left atrial and left ventricular pressures in subjects without cardiovascular disease: observations in eighteen patients studied by transseptal left heart catheterization. *Circulation*.

[19] Lasam G (2018). Brockenbrough-Braunwald-Morrow Sign: An Evaluative Hemodynamic Maneuver for Left Ventricular Outflow Tract Obstruction. *Cardiology Research*.

[20] Trevino AR, Buergler J (2014). The Brockenbrough-Braunwald-Morrow sign. *Methodist DeBakey Cardiovascular Journal*.

[21] Efthimiadis GK, Pagourelias ED, Parcharidou D, Gossios T, Kamperidis V, Theofilogiannakos EK (2013). Clinical characteristics and natural history of hypertrophic cardiomyopathy with midventricular obstruction. *Circulation Journal: Official Journal of the Japanese Circulation Society*.

[22] Takahashi H, Yamaguchi R, Ifuku M, Itaya M, Koga Y, Utsu F (1986). Left ventricular function in hypertrophic cardiomyopathy: a Tc-99m radionuclide angiographic study during exercise. *Journal of Cardiography. Supplement*.

[23] Olivotto I, Cecchi F, Poggesi C, Yacoub MH (2012). Patterns of disease progression in hypertrophic cardiomyopathy: an individualized approach to clinical staging. *Circulation. Heart Failure*.

[24] Harris KM, Spirito P, Maron MS, Zenovich AG, Formisano F, Lesser JR (2006). Prevalence, clinical profile, and significance of left ventricular remodeling in the end-stage phase of hypertrophic cardiomyopathy. *Circulation*.

[25] Zhang Y, Xie W, Dai Y, Wu Z, Lin Y, Yang M (2024). Influencing and prognostic factors of end-stage hypertrophic cardiomyopathy. *ESC Heart Failure*.

[26] Rowin EJ, Hausvater A, Link MS, Abt P, Gionfriddo W, Wang W (2017). Clinical Profile and Consequences of Atrial Fibrillation in Hypertrophic Cardiomyopathy. *Circulation*.

[27] Siontis KC, Geske JB, Ong K, Nishimura RA, Ommen SR, Gersh BJ (2014). Atrial fibrillation in hypertrophic cardiomyopathy: prevalence, clinical correlations, and mortality in a large high-risk population. *Journal of the American Heart Association*.

[28] Olivotto I, Cecchi F, Casey SA, Dolara A, Traverse JH, Maron BJ (2001). Impact of atrial fibrillation on the clinical course of hypertrophic cardiomyopathy. *Circulation*.

[29] Alphonse P, Virk S, Collins J, Campbell T, Thomas SP, Semsarian C (2021). Prognostic impact of atrial fibrillation in hypertrophic cardiomyopathy: a systematic review. *Clinical Research in Cardiology: Official Journal of the German Cardiac Society*.

[30] Rowin EJ, Link MS, Maron MS, Maron BJ (2023). Evolving Contemporary Management of Atrial Fibrillation in Hypertrophic Cardiomyopathy. *Circulation*.

[31] Zegkos T, Kamperidis V, Gossios T, Ntelios D, Parcharidou D, Papanastasiou CA (2022). Mitral regurgitation impact on left atrial myopathy in hypertrophic cardiomyopathy. *Echocardiography (Mount Kisco, N*.

[32] Debonnaire P, Joyce E, Hiemstra Y, Mertens BJ, Atsma DE, Schalij MJ (2017). Left Atrial Size and Function in Hypertrophic Cardiomyopathy Patients and Risk of New-Onset Atrial Fibrillation. *Circulation. Arrhythmia and Electrophysiology*.

[33] Guttmann OP, Pavlou M, O’Mahony C, Monserrat L, Anastasakis A, Rapezzi C (2017). Predictors of atrial fibrillation in hypertrophic cardiomyopathy. *Heart (British Cardiac Society)*.

[34] Hindricks G, Potpara T, Dagres N, Arbelo E, Bax JJ, Blomström-Lundqvist C (2021). 2020 ESC Guidelines for the diagnosis and management of atrial fibrillation developed in collaboration with the European Association for Cardio-Thoracic Surgery (EACTS): The Task Force for the diagnosis and management of atrial fibrillation of the European Society of Cardiology (ESC) Developed with the special contribution of the European Heart Rhythm Association (EHRA) of the ESC. *European Heart Journal*.

[35] Rozen G, Elbaz-Greener G, Marai I, Andria N, Hosseini SM, Biton Y (2020). Utilization and Complications of Catheter Ablation for Atrial Fibrillation in Patients With Hypertrophic Cardiomyopathy. *Journal of the American Heart Association*.

[36] Hodges K, Tang A, Rivas CG, Umana-Pizano J, Chemtob R, Desai MY (2020). Surgical ablation of atrial fibrillation in hypertrophic obstructive cardiomyopathy: Outcomes of a tailored surgical approach. *Journal of Cardiac Surgery*.

[37] Palandri C, Santini L, Argirò A, Margara F, Doste R, Bueno-Orovio A (2022). Pharmacological Management of Hypertrophic Cardiomyopathy: From Bench to Bedside. *Drugs*.

[38] Flamm MD, Harrison DC, Hancock EW (1968). Muscular subaortic stenosis. Prevention of outflow obstruction with propranolol. *Circulation*.

[39] Nistri S, Olivotto I, Maron MS, Ferrantini C, Coppini R, Grifoni C (2012). β Blockers for prevention of exercise-induced left ventricular outflow tract obstruction in patients with hypertrophic cardiomyopathy. *The American Journal of Cardiology*.

[40] Stenson RE, Flamm MD, Harrison DC, Hancock EW (1973). Hypertrophic subaortic stenosis. Clinical and hemodynamic effects of long-term propranolol therapy. *The American Journal of Cardiology*.

[41] Thompson DS, Naqvi N, Juul SM, Swanton RH, Coltart DJ, Jenkins BS (1980). Effects of propranolol on myocardial oxygen consumption, substrate extraction, and haemodynamics in hypertrophic obstructive cardiomyopathy. *British Heart Journal*.

[42] Dybro AM, Rasmussen TB, Nielsen RR, Ladefoged BT, Andersen MJ, Jensen MK (2022). Effects of Metoprolol on Exercise Hemodynamics in Patients With Obstructive Hypertrophic Cardiomyopathy. *Journal of the American College of Cardiology*.

[43] Doiuchi J, Hamada M, Ochi T, Ito T, Kokubu T (1985). Adverse effects of atrial fibrillation and syncope induced by calcium-channel blockers in hypertrophic cardiomyopathy. *Clinical Cardiology*.

[44] Bonow RO, Ostrow HG, Rosing DR, Cannon RO, Lipson LC, Maron BJ (1983). Effects of verapamil on left ventricular systolic and diastolic function in patients with hypertrophic cardiomyopathy: pressure-volume analysis with a nonimaging scintillation probe. *Circulation*.

[45] Hanrath P, Mathey DG, Kremer P, Sonntag F, Bleifeld W (1980). Effect of verapamil on left ventricular isovolumic relaxation time and regional left ventricular filling in hypertrophic cardiomyopathy. *The American Journal of Cardiology*.

[46] Anderson DM, Raff GL, Ports TA, Brundage BH, Parmley WW, Chatterjee K (1984). Hypertrophic obstructive cardiomyopathy. Effects of acute and chronic verapamil treatment on left ventricular systolic and diastolic function. *British Heart Journal*.

[47] Epstein SE, Rosing DR (1981). Verapamil: its potential for causing serious complications in patients with hypertrophic cardiomyopathy. *Circulation*.

[48] Betocchi S, Cannon RO, Watson RM, Bonow RO, Ostrow HG, Epstein SE (1985). Effects of sublingual nifedipine on hemodynamics and systolic and diastolic function in patients with hypertrophic cardiomyopathy. *Circulation*.

[49] Betocchi S, Piscione F, Losi M A, Pace L, Boccalatte M, Perrone-Filardi P (1996). Effects of diltiazem on left ventricular systolic and diastolic function in hypertrophic cardiomyopathy. *The American Journal of Cardiology*.

[50] Topriceanu CC, Field E, Boleti O, Cervi E, Kaski JP, Norrish G (2023). Disopyramide is a safe and effective treatment for children with obstructive hypertrophic cardiomyopathy. *International Journal of Cardiology*.

[51] Sherrid MV (2016). Drug Therapy for Hypertrophic Cardiomypathy: Physiology and Practice. *Current Cardiology Reviews*.

[52] Adler A, Fourey D, Weissler-Snir A, Hindieh W, Chan RH, Gollob MH (2017). Safety of Outpatient Initiation of Disopyramide for Obstructive Hypertrophic Cardiomyopathy Patients. *Journal of the American Heart Association*.

[53] Pollick C, Giacomini KM, Blaschke TF, Nelson WL, Turner-Tamiyasu K, Briskin V (1982). The cardiac effects of d- and l-disopyramide in normal subjects: a noninvasive study. *Circulation*.

[54] Coppini R, Ferrantini C, Pioner JM, Santini L, Wang ZJ, Palandri C (2019). Electrophysiological and Contractile Effects of Disopyramide in Patients With Obstructive Hypertrophic Cardiomyopathy: A Translational Study. *JACC. Basic to Translational Science*.

[55] Maurizi N, Chiriatti C, Fumagalli C, Targetti M, Passantino S, Antiochos P (2023). Real-World Use and Predictors of Response to Disopyramide in Patients with Obstructive Hypertrophic Cardiomyopathy. *Journal of Clinical Medicine*.

[56] Green EM, Wakimoto H, Anderson RL, Evanchik MJ, Gorham JM, Harrison BC (2016). A small-molecule inhibitor of sarcomere contractility suppresses hypertrophic cardiomyopathy in mice. *Science (New York, N.Y.)*.

[57] Olivotto I, Oreziak A, Barriales-Villa R, Abraham TP, Masri A, Garcia-Pavia P (2020). Mavacamten for treatment of symptomatic obstructive hypertrophic cardiomyopathy (EXPLORER-HCM): a randomised, double-blind, placebo-controlled, phase 3 trial. *Lancet (London, England)*.

[58] Hegde SM, Lester SJ, Solomon SD, Michels M, Elliott PM, Nagueh SF (2021). Effect of Mavacamten on Echocardiographic Features in Symptomatic Patients With Obstructive Hypertrophic Cardiomyopathy. *Journal of the American College of Cardiology*.

[59] Braunwald E, Saberi S, Abraham TP, Elliott PM, Olivotto I (2023). Mavacamten: a first-in-class myosin inhibitor for obstructive hypertrophic cardiomyopathy. *European Heart Journal*.

[60] Jaber WA, Nishimura RA, Ommen SR (2007). Not all systolic velocities indicate obstruction in hypertrophic cardiomyopathy: a simultaneous Doppler catheterization study. *Journal of the American Society of Echocardiography: Official Publication of the American Society of Echocardiography*.

[61] Geske JB, Cullen MW, Sorajja P, Ommen SR, Nishimura RA (2012). Assessment of left ventricular outflow gradient: hypertrophic cardiomyopathy versus aortic valvular stenosis. *JACC. Cardiovascular Interventions*.

[62] Elesber A, Nishimura RA, Rihal CS, Ommen SR, Schaff HV, Holmes DR (2008). Utility of isoproterenol to provoke outflow tract gradients in patients with hypertrophic cardiomyopathy. *The American Journal of Cardiology*.

[63] Cochran JM, Alam A, Guerrero-Miranda CY (2022). Importance of right heart catheterization in advanced heart failure management. *Reviews in Cardiovascular Medicine*.

[64] Present THE (2017). *Alcohol Septal Ablation for Obstructive*.

[65] Borlaug BA, Kass DA (2011). Invasive hemodynamic assessment in heart failure. *Cardiology Clinics*.

[66] Pinto YM, Elliott PM, Arbustini E, Adler Y, Anastasakis A, Böhm M (2016). Proposal for a revised definition of dilated cardiomyopathy, hypokinetic non-dilated cardiomyopathy, and its implications for clinical practice: a position statement of the ESC working group on myocardial and pericardial diseases. *European Heart Journal*.

[67] Berlin DA, Bakker J (2015). Starling curves and central venous pressure. *Critical Care (London, England)*.

[68] Moya A, Buytaert D, Penicka M, Bartunek J, Vanderheyden M (2023). State-of-the-Art: Noninvasive Assessment of Left Ventricular Function Through Myocardial Work. *Journal of the American Society of Echocardiography: Official Publication of the American Society of Echocardiography*.

[69] Gaasch WH, Meyer TE (2008). Left ventricular response to mitral regurgitation: implications for management. *Circulation*.

[70] Hasenfuss G, Mulieri LA, Leavitt BJ, Allen PD, Haeberle JR, Alpert NR (1992). Alteration of contractile function and excitation-contraction coupling in dilated cardiomyopathy. *Circulation Research*.

[71] Rodriguez J, Schulz S, Voss A, Herrera S, Benito S, Giraldo BF (2023). Baroreflex activity through the analysis of the cardio-respiratory variability influence over blood pressure in cardiomyopathy patients. *Frontiers in Physiology*.

[72] Norton JM (2001). Toward consistent definitions for preload and afterload. *Advances in Physiology Education*.

[73] Alter P, Rupp H, Rominger MB, Klose KJ, Maisch B (2008). A new methodological approach to assess cardiac work by pressure-volume and stress-length relations in patients with aortic valve stenosis and dilated cardiomyopathy. *Pflugers Archiv: European Journal of Physiology*.

[74] St Goar FG, Masuyama T, Alderman EL, Popp RL (1991). Left ventricular diastolic dysfunction in end-stage dilated cardiomyopathy: simultaneous Doppler echocardiography and hemodynamic evaluation. *Journal of the American Society of Echocardiography: Official Publication of the American Society of Echocardiography*.

[75] McDonagh TA, Metra M, Adamo M, Gardner RS, Baumbach A, Böhm M (2023). 2023 Focused Update of the 2021 ESC Guidelines for the diagnosis and treatment of acute and chronic heart failure. *European Heart Journal*.

[76] Merlo M, Caiffa T, Gobbo M, Adamo L, Sinagra G (2018). Reverse remodeling in Dilated Cardiomyopathy: Insights and future perspectives. *International Journal of Cardiology. Heart & Vasculature*.

[77] Heilbrunn SM, Shah P, Bristow MR, Valantine HA, Ginsburg R, Fowler MB (1989). Increased beta-receptor density and improved hemodynamic response to catecholamine stimulation during long-term metoprolol therapy in heart failure from dilated cardiomyopathy. *Circulation*.

[78] Andersson B, Hamm C, Persson S, Wikström G, Sinagra G, Hjalmarson A (1994). Improved exercise hemodynamic status in dilated cardiomyopathy after beta-adrenergic blockade treatment. *Journal of the American College of Cardiology*.

[79] Kim MH, Devlin WH, Das SK, Petrusha J, Montgomery D, Starling MR (1999). Effects of beta-adrenergic blocking therapy on left ventricular diastolic relaxation properties in patients with dilated cardiomyopathy. *Circulation*.

[80] Tong X, Shen L, Zhou X, Wang Y, Chang S, Lu S (2023). Comparative Efficacy of Different Drugs for the Treatment of Dilated Cardiomyopathy: A Systematic Review and Network Meta-analysis. *Drugs in R&D*.

[81] Pouleur H, Rousseau MF, van Eyll C, Stoleru L, Hayashida W, Udelson JA (1993). Effects of long-term enalapril therapy on left ventricular diastolic properties in patients with depressed ejection fraction. *SOLVD Investigators*.

[82] Hoshikawa E, Matsumura Y, Kubo T, Okawa M, Yamasaki N, Kitaoka H (2011). Effect of left ventricular reverse remodeling on long-term prognosis after therapy with angiotensin-converting enzyme inhibitors or angiotensin II receptor blockers and β blockers in patients with idiopathic dilated cardiomyopathy. *The American Journal of Cardiology*.

[83] Nikolaidis LA, Doverspike A, Huerbin R, Hentosz T, Shannon RP (2002). Angiotensin-converting enzyme inhibitors improve coronary flow reserve in dilated cardiomyopathy by a bradykinin-mediated, nitric oxide-dependent mechanism. *Circulation*.

[84] Lewis AB, Chabot M (1993). The effect of treatment with angiotensin-converting enzyme inhibitors on survival of pediatric patients with dilated cardiomyopathy. *Pediatric Cardiology*.

[85] Pitt B, Zannad F, Remme WJ, Cody R, Castaigne A, Perez A (2000). The Effect of Spironolactone on Morbidity and Mortality in Patients with Severe Heart Failure. *Survey of Anesthesiology*.

[86] Kasama S, Toyama T, Kumakura H, Takayama Y, Ichikawa S, Suzuki T (2003). Effect of spironolactone on cardiac sympathetic nerve activity and left ventricular remodeling in patients with dilated cardiomyopathy. *Journal of the American College of Cardiology*.

[87] Izawa H, Murohara T, Nagata K, Isobe S, Asano H, Amano T (2005). Mineralocorticoid receptor antagonism ameliorates left ventricular diastolic dysfunction and myocardial fibrosis in mildly symptomatic patients with idiopathic dilated cardiomyopathy: a pilot study. *Circulation*.

[88] Januzzi JL, Prescott MF, Butler J, Felker GM, Maisel AS, McCague K (2019). Association of Change in N-Terminal Pro-B-Type Natriuretic Peptide Following Initiation of Sacubitril-Valsartan Treatment With Cardiac Structure and Function in Patients With Heart Failure With Reduced Ejection Fraction. *JAMA*.

[89] Kim HM, Kim KH, Park JS, Oh BH (2021). Beneficial Effect of Left Ventricular Remodeling after Early Change of Sacubitril/Valsartan in Patients with Nonischemic Dilated Cardiomyopathy. *Medicina (Kaunas, Lithuania)*.

[90] Díez-Villanueva P, Vicent L, de la Cuerda F, Esteban-Fernández A, Gómez-Bueno M, de Juan-Bagudá J (2020). Left Ventricular Ejection Fraction Recovery in Patients with Heart Failure and Reduced Ejection Fraction Treated with Sacubitril/Valsartan. *Cardiology*.

[91] Vallon V, Verma S (2021). Effects of SGLT2 Inhibitors on Kidney and Cardiovascular Function. *Annual Review of Physiology*.

[92] Ji PJ, Zhang ZY, Yan Q, Cao HL, Zhao YJ, Yang B (2023). The cardiovascular effects of SGLT2 inhibitors, RAS inhibitors, and ARN inhibitors in heart failure. *ESC Heart Failure*.

[93] Hong J, Huang L, Jin N, Zhao X, Hu J (2024). Effect of dapagliflozin on left ventricular structure and function in patients with non-ischemic dilated cardiomyopathy: An observational study. *Medicine*.

[94] Psotka MA, Gottlieb SS, Francis GS, Allen LA, Teerlink JR, Adams KF (2019). Cardiac Calcitropes, Myotropes, and Mitotropes: JACC Review Topic of the Week. *Journal of the American College of Cardiology*.

[95] Malik FI, Hartman JJ, Elias KA, Morgan BP, Rodriguez H, Brejc K (2011). Cardiac myosin activation: a potential therapeutic approach for systolic heart failure. *Science (New York, N.Y.)*.

[96] Teerlink JR, Felker GM, McMurray JJV, Solomon SD, Adams KF, Cleland JGF (2016). Chronic Oral Study of Myosin Activation to Increase Contractility in Heart Failure (COSMIC-HF): a phase 2, pharmacokinetic, randomised, placebo-controlled trial. *Lancet (London, England)*.

[97] Rønning L, Bakkehaug JP, Rødland L, Kildal AB, Myrmel T, How OJ (2018). Opposite diastolic effects of omecamtiv mecarbil versus dobutamine and ivabradine co-treatment in pigs with acute ischemic heart failure. *Physiological Reports*.

[98] Teerlink JR, Diaz R, Felker GM, McMurray JJV, Metra M, Solomon SD (2021). Cardiac Myosin Activation with Omecamtiv Mecarbil in Systolic Heart Failure. *The New England Journal of Medicine*.

[99] Paulus WJ, Tschöpe C, Sanderson JE, Rusconi C, Flachskampf FA, Rademakers FE (2007). How to diagnose diastolic heart failure: a consensus statement on the diagnosis of heart failure with normal left ventricular ejection fraction by the Heart Failure and Echocardiography Associations of the European Society of Cardiology. *European Heart Journal*.

[100] Maeder MT, Kaye DM (2009). Heart failure with normal left ventricular ejection fraction. *Journal of the American College of Cardiology*.

[101] Nagueh SF, Smiseth OA, Appleton CP, Byrd BF, Dokainish H, Edvardsen T (2016). Recommendations for the Evaluation of Left Ventricular Diastolic Function by Echocardiography: An Update from the American Society of Echocardiography and the European Association of Cardiovascular Imaging. *Journal of the American Society of Echocardiography: Official Publication of the American Society of Echocardiography*.

[102] Yamamoto K, Nishimura RA, Redfield MM (1996). Assessment of mean left atrial pressure from the left ventricular pressure tracing in patients with cardiomyopathies. *The American Journal of Cardiology*.

[103] Haskell RJ, French WJ (1988). Accuracy of left atrial and pulmonary artery wedge pressure in pure mitral regurgitation in predicting left ventricular end-diastolic pressure. *The American Journal of Cardiology*.

[104] Riccardi M, Pagnesi M, Sciatti E, Lombardi CM, Inciardi RM, Metra M (2023). Combined pre- and post-capillary pulmonary hypertension in left heart disease. *Heart Failure Reviews*.

[105] Chaudhry A, Singer AJ, Chohan J, Russo V, Lee C (2008). Interrater reliability of hemodynamic profiling of patients with heart failure in the ED. *The American Journal of Emergency Medicine*.

[106] Costanzo-Nordin MR, O’Connell JB, Engelmeier RS, Moran JF, Scanlon PJ (1985). Dilated cardiomyopathy: functional status, hemodynamics, arrhythmias, and prognosis. *Catheterization and Cardiovascular Diagnosis*.

[107] Pinamonti B, Zecchin M, Di Lenarda A, Gregori D, Sinagra G, Camerini F (1997). Persistence of restrictive left ventricular filling pattern in dilated cardiomyopathy: an ominous prognostic sign. *Journal of the American College of Cardiology*.

[108] Stolfo D, Merlo M, Pinamonti B, Poli S, Gigli M, Barbati G (2015). Early improvement of functional mitral regurgitation in patients with idiopathic dilated cardiomyopathy. *The American Journal of Cardiology*.

[109] Japp AG, Gulati A, Cook SA, Cowie MR, Prasad SK (2016). The Diagnosis and Evaluation of Dilated Cardiomyopathy. *Journal of the American College of Cardiology*.

[110] Kažukauskienė I, Balčiūnaitė G, Baltrūnienė V, Čelutkienė J, Maneikienė VV, Čibiras S (2021). Left ventricular global longitudinal strain predicts elevated cardiac pressures and poor clinical outcomes in patients with non-ischemic dilated cardiomyopathy. *Cardiovascular Ultrasound*.

[111] Ciarambino T, Menna G, Sansone G, Giordano M (2021). Cardiomyopathies: An Overview. *International Journal of Molecular Sciences*.

[112] Rapezzi C, Aimo A, Barison A, Emdin M, Porcari A, Linhart A (2022). Restrictive cardiomyopathy: definition and diagnosis. *European Heart Journal*.

[113] Mogensen J, van Tintelen JP, Fokstuen S, Elliott P, van Langen IM, Meder B (2015). The current role of next-generation DNA sequencing in routine care of patients with hereditary cardiovascular conditions: a viewpoint paper of the European Society of Cardiology working group on myocardial and pericardial diseases and members of the European Society of Human Genetics. *European Heart Journal*.

[114] Kaski JP, Syrris P, Burch M, Tomé-Esteban MT, Fenton M, Christiansen M (2008). Idiopathic restrictive cardiomyopathy in children is caused by mutations in cardiac sarcomere protein genes. *Heart (British Cardiac Society)*.

[115] Goldstein JA, Kern MJ (2020). Hemodynamics of constrictive pericarditis and restrictive cardiomyopathy. *Catheterization and Cardiovascular Interventions: Official Journal of the Society for Cardiac Angiography & Interventions*.

[116] Rapezzi C, Merlini G, Quarta CC, Riva L, Longhi S, Leone O (2009). Systemic cardiac amyloidoses: disease profiles and clinical courses of the 3 main types. *Circulation*.

[117] Sanchorawala V (2024). Systemic Light Chain Amyloidosis. *The New England Journal of Medicine*.

[118] Maurer MS, Schwartz JH, Gundapaneni B, Elliott PM, Merlini G, Waddington-Cruz M (2018). Tafamidis Treatment for Patients with Transthyretin Amyloid Cardiomyopathy. *The New England Journal of Medicine*.

[119] Gillmore JD, Judge DP, Cappelli F, Fontana M, Garcia-Pavia P, Gibbs S (2024). Efficacy and Safety of Acoramidis in Transthyretin Amyloid Cardiomyopathy. *The New England Journal of Medicine*.

[120] Adams D, Gonzalez-Duarte A, O’Riordan WD, Yang CC, Ueda M, Kristen AV (2018). Patisiran, an RNAi Therapeutic, for Hereditary Transthyretin Amyloidosis. *The New England Journal of Medicine*.

[121] Maurer MS, Kale P, Fontana M, Berk JL, Grogan M, Gustafsson F (2023). Patisiran Treatment in Patients with Transthyretin Cardiac Amyloidosis. *The New England Journal of Medicine*.

[122] Coelho T, Marques W, Dasgupta NR, Chao CC, Parman Y, França MC (2023). Eplontersen for Hereditary Transthyretin Amyloidosis With Polyneuropathy. *JAMA*.

[123] Brannagan TH, Coelho T, Wang AK, Polydefkis MJ, Dyck PJ, Berk JL (2022). Long-term efficacy and safety of inotersen for hereditary transthyretin amyloidosis: NEURO-TTR open-label extension 3-year update. *Journal of Neurology*.

[124] Gillmore JD, Gane E, Taubel J, Kao J, Fontana M, Maitland ML (2021). CRISPR-Cas9 In Vivo Gene Editing for Transthyretin Amyloidosis. *The New England Journal of Medicine*.

[125] Pieroni M, Moon JC, Arbustini E, Barriales-Villa R, Camporeale A, Vujkovac AC (2021). Cardiac Involvement in Fabry Disease: JACC Review Topic of the Week. *Journal of the American College of Cardiology*.

[126] Weidemann F, Niemann M, Breunig F, Herrmann S, Beer M, Störk S (2009). Long-term effects of enzyme replacement therapy on fabry cardiomyopathy: evidence for a better outcome with early treatment. *Circulation*.

[127] Weidemann F, Jovanovic A, Herrmann K, Vardarli I (2022). Chaperone Therapy in Fabry Disease. *International Journal of Molecular Sciences*.

[128] Vardarli I, Weber M, Rischpler C, Führer D, Herrmann K, Weidemann F (2021). Fabry Cardiomyopathy: Current Treatment and Future Options. *Journal of Clinical Medicine*.

[129] Domm JM, Wootton SK, Medin JA, West ML (2021). Gene therapy for Fabry disease: Progress, challenges, and outlooks on gene-editing. *Molecular Genetics and Metabolism*.

[130] Kang Y, Qu N, Zhang Z, Zhang Q, Chen X, Fu M (2024). Tolerability and effectiveness of beta-blockers in patients with cardiac amyloidosis: A systematic review and meta-analysis. *International Journal of Cardiology*.

[131] Muchtar E, Blauwet LA, Gertz MA (2017). Restrictive Cardiomyopathy: Genetics, Pathogenesis, Clinical Manifestations, Diagnosis, and Therapy. *Circulation Research*.

[132] Bukhari S, Oliveros E, Parekh H, Farmakis D (2023). Epidemiology, Mechanisms, and Management of Atrial Fibrillation in Cardiac Amyloidosis. *Current Problems in Cardiology*.

[133] Redfield MM, Borlaug BA (2023). Heart Failure With Preserved Ejection Fraction: A Review. *JAMA*.

[134] Felker GM, Ellison DH, Mullens W, Cox ZL, Testani JM (2020). Diuretic Therapy for Patients With Heart Failure: JACC State-of-the-Art Review. *Journal of the American College of Cardiology*.

[135] Packer M, Butler J, Zannad F, Filippatos G, Ferreira JP, Pocock SJ (2021). Effect of Empagliflozin on Worsening Heart Failure Events in Patients With Heart Failure and Preserved Ejection Fraction: EMPEROR-Preserved Trial. *Circulation*.

[136] Peikert A, Martinez FA, Vaduganathan M, Claggett BL, Kulac IJ, Desai AS (2022). Efficacy and Safety of Dapagliflozin in Heart Failure With Mildly Reduced or Preserved Ejection Fraction According to Age: The DELIVER Trial. *Circulation. Heart Failure*.

[137] Verma A, Patel AB, Waikar SS (2020). SGLT2 Inhibitor: Not a Traditional Diuretic for Heart Failure. *Cell Metabolism*.

[138] Lopaschuk GD, Verma S (2020). Mechanisms of Cardiovascular Benefits of Sodium Glucose Co-Transporter 2 (SGLT2) Inhibitors: A State-of-the-Art Review. *JACC. Basic to Translational Science*.

[139] Nakatsukasa T, Ishizu T, Ouchi M, Murakoshi N, Sato K, Yamamoto M (2022). Sodium Glucose Co-Transporter 2 Inhibitors Improve Renal Congestion and Left Ventricular Fibrosis in Rats With Hypertensive Heart Failure. *Circulation Journal: Official Journal of the Japanese Circulation Society*.

[140] Garcia MJ (2016). Constrictive Pericarditis Versus Restrictive Cardiomyopathy?. *Journal of the American College of Cardiology*.

[141] Geske JB, Anavekar NS, Nishimura RA, Oh JK, Gersh BJ (2016). Differentiation of Constriction and Restriction: Complex Cardiovascular Hemodynamics. *Journal of the American College of Cardiology*.

[142] Pereira NL, Grogan M, Dec GW (2018). Spectrum of Restrictive and Infiltrative Cardiomyopathies: Part 1 of a 2-Part Series. *Journal of the American College of Cardiology*.

